# Caffeine Increases Apolipoprotein A-1 and Paraoxonase-1 but not Paraoxonase-3 Protein Levels in Human-Derived Liver (HepG2) Cells

**DOI:** 10.4274/balkanmedj.2016.1217

**Published:** 2017-12-01

**Authors:** Gülben Sayılan Özgün, Eray Özgün, Kıymet Tabakçıoğlu, Selma Süer Gökmen, Sevgi Eskiocak, Erol Çakır

**Affiliations:** 1 Department of Medical Biochemistry, Trakya University School of Medicine, Edirne, Turkey; 2 Department of Medical Biology, Trakya University School of Medicine, Edirne, Turkey

**Keywords:** Caffeine, apolipoprotein A-1, paraoxonase-1, paraoxonase-3, HepG2 cells

## Abstract

**Background:**

Apolipoprotein A-1, paraoxonase-1 and paraoxonase-3 are antioxidant and anti-atherosclerotic structural high-density lipoprotein proteins that are mainly synthesized by the liver. No study has ever been performed to specifically examine the effects of caffeine on paraoxonase enzymes and on liver apolipoprotein A-1 protein levels.

**Aims:**

To investigate the dose-dependent effects of caffeine on liver apolipoprotein A-1, paraoxonase-1 and paraoxonase-3 protein levels.

**Study Design:**

*In vitro* experimental study.

**Methods:**

HepG2 cells were incubated with 0 (control), 10, 50 and 200 μM of caffeine for 24 hours. Cell viability was evaluated by 3-(4,5-Dimethyl-2-thiazolyl)-2,5-diphenyl-2H-tetrazolium bromide assay. Apolipoprotein A-1, paraoxonase-1 and paraoxonase-3 protein levels were measured by western blotting.

**Results:**

We observed a significant increase on apolipoprotein A-1 and paraoxonase-1 protein levels in the cells incubated with 50 µM of caffeine and a significant increase on paraoxonase-1 protein level in the cells incubated with 200 µM of caffeine.

**Conclusion:**

Our study showed that caffeine does not change paraoxonase-3 protein level, but the higher doses used in our study do cause an increase in both apolipoprotein A-1 and paraoxonase-1 protein levels in liver cells.

Although the pathogenesis of atherosclerosis is multifactorial, clinical and epidemiological studies show that increased low-density lipoprotein (LDL) cholesterol levels and decreased high-density lipoprotein (HDL) levels contribute significantly to the development and progression of cardiovascular disease. However, growing evidence also indicates that oxidative modifications of LDL, which depend on the balance between pro-oxidants and antioxidants, play a vital role in the pathogenesis of atherosclerosis (1). LDL is protected from oxidation primarily by endogenous antioxidants. Antioxidants prevent atherosclerosis interacting directly with LDL or indirectly with cellular oxidative mechanisms. It has been reported that dietary antioxidants can decrease cell-mediated oxidation and, therefore, prevent the development of atherosclerosis by inhibiting LDL oxidation (2,3).

Apolipoprotein A-1 (ApoA1), mainly synthesised by the liver, is the major structural and functional HDL protein. ApoA1 is an anti-atherogenic and antioxidant protein. It plays an important role in reverse cholesterol transport and protects LDL against oxidation (4). Paraoxonase (PON)-1 and PON3 are antioxidant enzymes that are mainly synthesised by the liver, and they are bound to HDL in serum (5). PON1 prevents LDL oxidation and foam-cell formation, thereby inhibiting atherosclerosis development (6). Also, PON3 prevents mildly oxidized LDL formation and, therefore, monocyte chemotactic activity (7,8). Oxidized LDL, in turn, can inactivate PON activity (2).

It has been shown that ApoA1, PON1 and PON3 are antioxidant, anti-inflammatory and anti-atherosclerotic proteins, and they play important roles in the atheroprotective activities of HDL (4,5). A large number of studies have reported that dietary antioxidants may decrease the development of atherosclerosis. Possible mechanisms of dietary antioxidants include inhibition of LDL oxidation and preservation or increase of PON enzyme activities (3).

Caffeine, a methylxanthine, is the most widely consumed substance in the human diet. This psychoactive alkaloid is found primarily in the seeds, nuts, and leaves of some plants. It is also readily available in a variety of foods and beverages that we consume in our daily lives, such as chocolate, coffee, tea and energy drinks (9). Well-known effects of caffeine on the body include increased motor and mental activities through the stimulation of the central nervous system, increased heart rate and diuresis. Caffeine is used to mitigate sleepiness, to suppress appetite, to treat apnoea in premature infants, to enhance performance and for headache therapy (10).

Despite several previous reports of the effect of caffeine on atherosclerosis (11,12,13), no study has ever been performed to specifically examine the effects of caffeine on PON enzymes. Although the effect of caffeine on serum ApoA1 levels has been reported by some investigators (14,15,16), we have not encountered any studies that investigate the effects of caffeine on liver ApoA1 protein levels. The aim of the present study was to investigate the dose-dependent effects of caffeine on ApoA1, PON1 and PON3 protein levels in the liver.

Because ApoA1, PON1 and PON3 are primarily synthesised by the liver, we chose human-derived liver (HepG2) cells. HepG2 cells are useful tools in the understanding of hepatic protein biosynthesis and retain many biological characteristics of hepatocytes (17). Previous studies also used HepG2 cells to investigate ApoA1, PON1 and PON3 expressions in the liver (8,18).

It has been reported that plasma caffeine concentrations are usually between 2-10 mg/L (approximately 10-50 μM) in adults, depending on daily coffee consumption. Toxic effects are observed at a plasma caffeine concentration higher than 40 mg/L (approximately 200 μM) (19,20). Therefore, for this study we investigated the effects of caffeine concentrations at 10, 50 and 200 μM.

## MATERIALS AND METHODS

### Chemicals

Human HepG2 cells were purchased from ATCC (Middlesex, UK). Caffeine was purchased from Sigma-Aldrich Co. (St. Louis, MO, USA). Minimum essential medium (MEM) with glutamine, fetal bovine serum, antibiotic-antimycotic, sodium pyruvate, trypsin-ethylenediaminetetraacetic acid and horseradish peroxidase (HRP) chemiluminescent substrate were purchased from Thermo Fisher (Waltham, MA USA). PON1, PON3, alpha tubulin primary antibodies and goat anti-mouse IgG H&L HRP secondary antibody were purchased from Abcam (Cambridge, UK). ApoA1 primary antibody was purchased from Novus Biologicals Inc. (Littleton, CO, USA). The radioimmunoprecipitation assay (RIPA) lysis buffer system was purchased from Santa Cruz (Heidelberg, Germany). The polyvinylidene fluoride (PVDF) membrane was purchased from Bio-Rad (Hercules, CA, USA). Other chemicals were purchased from Sigma-Aldrich Co. (St. Louis, MO, USA) or Merck (Darmstadt, Germany). All reagents were of analytical grade.

### Cell culture and experimental design

This study was approved by the ethics committee of Trakya University School of Medicine (TÜTF-GOKAEK 2014/123, date of approval: 25 June 2014). HepG2 cells were cultured in MEM with glutamine containing 10% foetal bovine serum, 1% sodium pyruvate and 1% antibiotic-antimycotic (Thermo Fisher catalog number: 15240062) in a humidified environment at 37 °C and a 5% CO_2_ atmosphere.

HepG2 cells were divided into four groups; control cells (cultured in medium without caffeine for 24 hours), 10 μM caffeine-treated cells (cultured with 10 μM of caffeine for 24 hours), 50 μM caffeine-treated cells (cultured with 50 μM of caffeine for 24 hours) and 200 μM caffeine-treated cells (cultured with 200 μM of caffeine for 24 hours). For each group, the caffeine was dissolved in media. All of the experiments were repeated at least three times.

### Cell viability assays

The effect of caffeine on cell viability was evaluated using the 3-(4,5-Dimethyl-2-thiazolyl)-2,5-diphenyl-2H-tetrazolium bromide (MTT) assay (21). 1.5x104 cells were seeded into 96-well plates. Cells were treated with caffeine (10, 50 and 200 mM) for 24 hrs. At the end of treatment, media were removed and 10 µL of MTT (5 mg/mL) were dissolved in phosphate-buffered saline, and 100 µL of medium without phenol red were then added to each well. Cells were then incubated for 4 hrs in a humidified environment at 37 °C and a 5% CO_2_ atmosphere. MTT-containing medium was then removed. Formazan crystals, formed by MTT reduction, were dissolved using 200 µL dimethyl sulfoxide and 25 µL Sorensen buffer (0.1 M glycine, 0.1 M sodium chloride, pH 10.5). The optical density of plates was measured using a microplate reader at 570/630 nm. The optical density of each sample was then compared with the mean optical density value of the control group.

### Western blot analysis of PON1 and PON3 proteins

Cells were seeded into a 75 cm^2^ flask. After the cells reached 70-80% confluence, they were treated with caffeine (10, 50 and 200 mM) for 24 hours. Following the treatments, cells were scraped with RIPA lysis buffer system. Samples *were homogenized and then centrifuged* at 4 °C for 10 minutes at 15.000×g. Supernatants were used for protein determination and western blotting. Protein concentrations were measured according to Lowry et al. (22).

10 mg total protein for PON1 and 30 mg total protein for PON3 and ApoA1 were separated by sodium dodecyl sulfate-polyacyrlamide gel (4-8%) electrophoresis. With a semi-dry blotting system, proteins were transferred to a PVDF membrane. Membranes were blocked by incubating them with skim milk powder (5%) for 1 hour at room temperature. After blocking, membranes were incubated with monoclonal primary antibodies (PON1: 1/500 dilution, PON3: 1/2.000 dilution and ApoA1: 1/1.000 dilution) overnight at 4 °C and then with secondary antibody (HRP goat anti-mouse: 1/10.000 dilution) at room temperature for one hour. Protein bands were visualised by using an electrochemiluminescence (ECL) detection system with an HRP chemiluminescent substrate, and the bands were quantified using Image J (23). Results were calculated relative to alpha-tubulin as the loading control, and they were expressed as fold changes relative to the control for each blot.

### Statistical analysis

Results were given as means ± standard deviation. The one-way analysis of variance (ANOVA) test was used to compare the biochemical parameters among the groups. The Tamhane post-hoc test was used for multiple comparisons when a significant difference was obtained with the one-way ANOVA. SPSS 20.0 (IBM SPSS Inc., Chicago, IL, USA) statistical software was used for the statistical analysis; a p<0.05 was considered statistically significant.

## RESULTS

**FIG. 1.**
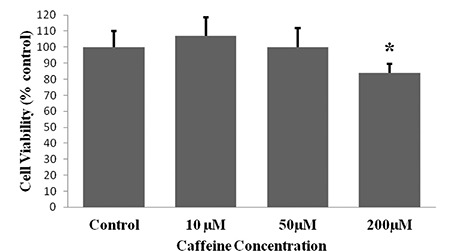
The cell viabilities of HepG2 cells after incubation with caffeine for 24 hours caffeine. Results are expressed as the mean ± standard deviation for each caffeine concentration (n=24 for all concentrations). The effect of caffeine was analysed by One-way analysis of variance. A Tamhane test was performed for multiple comparisons between experimental groups. *p<0.001, compared with control, 10 μM and 50 μM caffeine-treated cells.

**FIG. 2.**
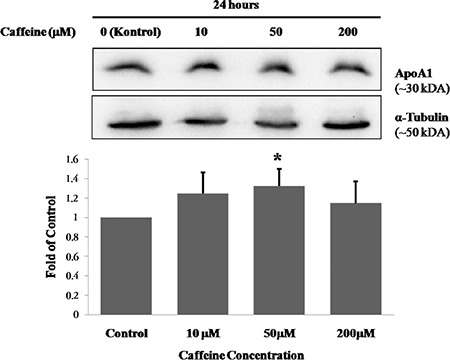
The effect of caffeine on ApoA1 protein levels in HepG2 cells. Results are expressed as the mean ± standard deviation for each caffeine concentration (n=6 for all concentrations). The effect of caffeine was analysed by One-way analysis of variance. A Tamhane test was performed for multiple comparisons between experimental groups. *p<0.05, compared with control.

**FIG. 3.**
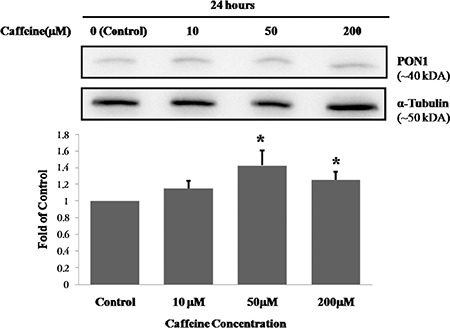
The effect of caffeine on PON1 protein levels in HepG2 cells. Results are expressed as the mean ± standard deviation for each caffeine concentration (n=6 for all concentrations). The effect of caffeine was analysed by one-way analysis of variance. A Tamhane test was performed for multiple comparisons between experimental groups. *p<0.05, compared with control.

**FIG. 4.**
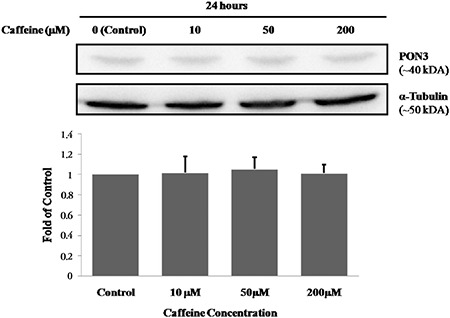
The effect of caffeine on PON3 protein levels in HepG2 cells. Results are expressed as the mean ± standard deviation for each caffeine concentration (n=6 for all concentrations). The effect of caffeine was analysed by one-way analysis of variance. p>0.05 between caffeine concentrations.

The mean percentage of cell viabilities of caffeine-treated groups was 107% for 10 µM caffeine-treated cells, 100% for 50 µM caffeine-treated cells and 84% for 200 µM caffeine-treated cells. Cell viability in those treated with 200 µM of caffeine was significantly lower than other groups (p<0.001 for all) (Figure 1). ApoA1 protein levels of the caffeine-treated groups were 1.25-fold, 1.32-fold and 1.15-fold higher for the cells exposed to 10, 50 and 200 µM of caffeine, respectively. The ApoA1 protein levels in the 50 µM caffeine-treated cells were significantly increased compared to those in the control cells (p<0.05 for both) (Table 1, Figure 2). The PON1 protein levels of the caffeine-treated cells were 1.15-fold, 1.43-fold and 1.25-fold higher for the cells exposed to 10, 50 and 200 µM of caffeine, respectively. The PON1 protein levels in the 50 and 200 µM caffeine-treated cells were significantly increased compared to the protein levels in the control cells (p<0.05 for both) (Table 1, Figure 3). The PON3 protein levels of the caffeine-treated groups were 1.02-fold, 1.05-fold and 1.01-fold higher for the cells exposed to 10, 50 and 200 µM of caffeine, respectively. There were no significant differences between PON3 protein levels for any of the groups (p>0.05 for all) (Table 1, Figure 4).

## DISCUSSION

Caffeine has a variety of pharmacological and cellular responses in biological systems. Some well-known effects of caffeine include central nervous system and cardiac muscle stimulation, diuresis and smooth muscle relaxation (10). It is known that caffeine has several mechanisms of action. The most prominent one is reversibly binding to the adenosine receptor to block its action. Caffeine promotes wakefulness by this mechanism (10,24). Azam et al. (25) also reported that caffeine exhibits both antioxidant and pro-oxidant properties.

LDL oxidation is an early event in atherosclerosis, which is a major cause of mortality in developed countries. Dietary antioxidants can inhibit the oxidative modification of LDL, so they have the potential to decrease the risk of developing atherosclerosis (3). It has also been reported that dietary antioxidants may reduce atherogenesis by preservation or increase of PON activity of HDL (3).

Contradictory results have been reported on the relationship between caffeine and atherosclerosis risk. Papamichael et al. (12) reported that coffee, due to its caffeine content, exerts an acute unfavourable effect on endothelial function in healthy individuals. Lane et al. (11) reported that daily caffeine intake increases total blood cholesterol and LDL cholesterol levels, but decreases HDL cholesterol levels. It has been reported that caffeine does not change serum ApoA1 levels (14), and there is no association between caffeine intake and coronary and carotid atherosclerosis (13). On the other hand, it has been reported that caffeine increases serum ApoA1 levels, which is the major structural and functional protein of HDL (15,16). Yukawa et al. (26) also reported that coffee ingestion resulted in a decrease in total serum cholesterol, LDL cholesterol and malondialdehyde levels and in the susceptibility of LDL to oxidation.

Taking into consideration these studies, in the present study we examined the dose-dependent effects of caffeine on liver PON1, PON3 and ApoA1 protein levels, and we evaluated a possible relationship between caffeine and atherosclerosis. We incubated HepG2 cells with 0 (control), 10, 50 and 200 μM of caffeine for 24 h and found that the cell viability of cells treated with 200 μM of caffeine was significantly lower than other groups. Our findings are in agreement with another study that reported toxic effects of caffeine at plasma concentrations of caffeine that were higher than 200 μM (19).

For the first time, we investigated the effects of caffeine on liver ApoA1 protein levels and found that 50 mM of caffeine significantly increased ApoA1 protein level in HepG2 cells. This finding is supported by previous studies that reported increases in serum ApoA1 levels caused by caffeine (15,16).

Our study is also the first report to investigate the effects of caffeine on the PON1 enzyme. In this study, we found that both 50 mM and 200 mM of caffeine significantly increased PON1 protein levels in HepG2 cells. Other studies have reported that PON1 protein levels and activity are decreased in ApoA1 knock-out mice (27), and the overexpression of ApoA1 increases PON1 activity in transgenic mice (28). The regulation of PON1 enzyme is linked to HDL and ApoA1 modulation (5). The findings reported in these studies are supported by the findings of our study, in which 50 mM of caffeine significantly increased PON1 protein and ApoA1 levels. A plasma concentration of 50 mM of caffeine, which is equal to the average plasma caffeine concentration of healthy individuals who consume coffee, may decrease the risk of atherosclerosis by increasing liver ApoA1 and PON1 protein levels.

In the present study, 200 mM of caffeine, which is the plasma concentration of caffeine at which toxicity has been observed, decreased cell viability but increased PON1 protein level of HepG2 cells; this was a surprising result. Caffeine has been reported to exhibit both antioxidant and pro-oxidant properties (25). Ikeda et al. (29) reported that high glucose induces PON1 protein levels in cultured hepatocytes, and that increasing PON1 protein levels may be a compensatory mechanism in diabetes. Our results, which were similar to those of Ikeda et al. (29), suggest that an increase in PON1 protein levels in cells treated with 200 μM of caffeine may be a cell’s defence mechanism against caffeine toxicity in the liver.

In the present study, the effects of caffeine on the PON3 enzyme were also investigated for the first time. We demonstrated that exposure to caffeine does not significantly change PON3 protein levels at non-toxic (10 and 50 μM) or toxic (200 μM) plasma concentrations. This finding indicates that caffeine may not have an effect on the regulation of PON3 protein level. This finding is important because it indicates that the regulation of the PON3 enzyme may be different from that of the PON1 enzyme, even though they are members of the same gene family. This finding is in agreement with the finding of Reddy et al. (8), who reported that while PON1 is regulated by oxidized lipids, PON3 is not regulated by oxidized lipids. It is also in agreement with our previous study, which indicated that PON3 is more sensitive to lipoic acid than PON1 in diabetic rats (30). Further research should address these mechanisms.

In conclusion, our study showed that caffeine does not change PON3 protein levels, but it increases both ApoA1 and PON1 protein levels in liver cells. According to our results, caffeine may reduce atherosclerosis risk by increasing liver ApoA1 and PON1 protein levels. A limitation of this study is that we used an immortal hepatoma cell line instead of hepatocytes. Also, we only investigated ApoA1, PON1 and PON3 enzymes in liver cells; we did not consider other factors that are related to atherosclerosis in the liver or in other tissues such as endothelium and blood. Therefore, this *in vitro* study needs to be supported by future animal and human studies.

## Figures and Tables

**Table 1:**
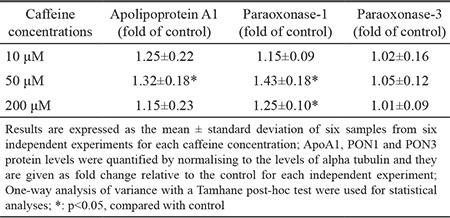
ApoA1, PON1 and PON3 protein levels following exposure to different concentrations of caffeine

## References

[1] Singh U, Jialal I (2006). Oxidative stres and atherosclerosis. Pathophysiology.

[2] Aviram M (1999). Macrophage foam cell formation during early atherogenesis is determined by the balance between pro-oxidants and anti-oxidants in arterial cells and blood lipoproteins. Antioxid Redox Signal.

[3] Aviram M, Kaplan M, Rosenblat M, Fuhrman B (2005). Dietary antioxidants and paraoxonases against LDL oxidation and atherosclerosis development. Handb Exp Pharmacol.

[4] Podrez EA (2010). Anti-oxidant properties of high-density lipoprotein and atherosclerosis. Clin Exp Pharmacol Physiol.

[5] Précourt LP, Amre D, Denis MC, Lavoie JC, Delvin E, Seidman E, et al (2011). The three-gene paraoxonase family: physiologic roles, actions and regulation. Atherosclerosis.

[6] Aviram M, Rosenblat M (2004). Paraoxonases 1, 2, and 3, oxidative stress, and macrophage foam cell formation during atherosclerosis development. Free Radic Biol Med.

[7] Draganov DI, Stetson PL, Watson CE, Billecke SS, La Du BN (2000). Rabbit serum paraoxonase 3 (PON3) is a high density lipoprotein-associated lactonase and protects low density lipoprotein against oxidation. J Biol Chem.

[8] Reddy ST, Wadleigh DJ, Grijalva V, Ng C, Hama S, Gangopadhyay A, et al (2001). Human paraoxonase-3 is an HDL-associated enzyme with biological activity similar to paraoxonase-1 protein but is not regulated by oxidized lipids. Arterioscler Thromb Vasc Biol.

[9] Clark I, Landolt HP (2016). Coffee, caffeine, and sleep: A systematic review of epidemiological studies and randomized controlled trials. Sleep Med Rev.

[10] Winston AP, Hardwick E, Jaberi N (2005). Neuropsychiatric effects of caffeine. Adv Psychiatr Treat.

[11] Lane JD, Pieper CF, Barefoot JC, Williams RB Jr, Siegler IC (1994). Caffeine and cholesterol: interactions with hostility. Psychosom Med.

[12] Papamichael CM, Aznaouridis KA, Karatzis EN, Karatzi KN, Stamatelopoulos KS, Vamvakou G, et al (2005). Effect of coffee on endothelial function in healthy subjects: the role of caffeine. Clin Sci (Lond).

[13] Reis JP, Loria CM, Steffen LM, Zhou X, van Horn L, Siscovick DS, et al (2010). Coffee, decaffeinated coffee, caffeine, and tea consumption in young adulthood and atherosclerosis later in life: the CARDIA study. Arterioscler Thromb Vasc Biol.

[14] Sedor FA, Schneider KA, Heyden S (1991). Effect of coffee on cholesterol and apolipoproteins, corroborated by caffeine levels. Am J Prev Med.

[15] Carson CA, Cauley JA, Caggiula AW (1993). Relation of caffeine intake to blood lipids in elderly women. Am J Epidemiol.

[16] Kempf K, Herder C, Erlund I, Kolb H, Martin S, Carstensen M, et al (2010). Effects of coffee consumption on subclinical inflammation and other risk factors for type 2 diabetes: a clinical trial. Am J Clin Nutr.

[17] Bouma ME, Rogier E, Verthier N, Labarre C, Feldmann G (1989). Further cellular investigation of the human hepatoblastoma-derived cell line HepG2: morphology and immunocytochemical studies of hepatic-secreted proteins. In Vitro Cell Dev Biol.

[18] Jaichander P, Selvarajan K, Garelnabi M, Parthasarathy S (2008). Induction of paraoxonase 1 and apolipoprotein A-I gene expression by aspirin. J Lipid Res.

[19] Kulkarni PB, Dorand RD (1979). Caffeine toxicity in a neonate. Pediatrics.

[20] Fredholm BB, Bättig K, Holmén J, Nehlig A, Zvartau EE (1999). Actions of caffeine in the brain with special reference to factors that contribute to its widespread use. Pharmacol Rev.

[21] Mosmann T, Mosmann T (1983). Rapid colorimetric assay for cellular growth and survival: application to proliferation and cytotoxicity assays. J Immunol Methods.

[22] Lowry OH, Rosebrough NJ, Farr AL, Randall RJ (1951). Protein measurement with the Folin phenol reagent. J Biol Chem.

[23] Schneider CA, Rasband WS, Eliceiri KW (2012). NIH Image to ImageJ: 25 years of image analysis. Nat Methods.

[24] Nehlig A, Daval JL, Debry G (1992). Caffeine and the central nervous system: mechanisms of action, biochemical, metabolic and psychostimulant effects. Brain Res Rev.

[25] Azam S, Hadi N, Khan NU, Hadi SM (2003). Antioxidant and prooxidant properties of caffeine, theobromine and xanthine. Med Sci Monit.

[26] Yukawa GS, une M, Otani H, Tone Y, Liang XM, Iwahashi H, et al (2004). Effects of coffee consumption on oxidative susceptibility of low-density lipoproteins and serum lipid levels in humans. Biochemistry (Mosc).

[27] Moore RE, Navab M, Millar JS, Zimetti F, Hama S, Rothblat GH, et al (2005). Increased atherosclerosis in mice lacking apolipoprotein A-I attributable to both impaired reverse cholesterol transport and increased inflammation. Circ Res.

[28] De Geesr B, Stengel D, Landeloos M, Lox M, e Gat L, Collen D, et al (2000). Effect of overexpression of human apo A-I in C57BL/6 and C57BL/6 apo E-deficient mice on 2 lipoprotein-associated enzymes, platelet-activating factor acetylhydrolase and paraoxonase. Comparison of adenovirus-mediated human apo A-I gene transfer and human apo A-I transgenesis. Arterioscler Thromb Vasc Biol.

[29] Ikeda Y, Suehiro T, Arii K, Kumon Y, Hashimoto K (2008). High glucose induces transactivation of the human paraoxonase 1 gene in hepatocytes. Metabolism.

[30] Ozgun E, Ozgun GS, Gokmen SS, Eskiocak S, Sut N, Akıncı M, et al (2016). Effect of lipoic acid on serum paraoxonase-1 and paraoxonase-3 protein levels and activities in diabetic rats. Exp Clin Endocrinol Diabetes.

